# Nitric oxide activity-dependent regulator compensates synaptic depression and enhances metabolic efficiency in the auditory brainstem

**DOI:** 10.1186/1471-2202-15-S1-P154

**Published:** 2014-07-21

**Authors:** Christophe B Michel, Matthias H Hennig, Bruce P Graham

**Affiliations:** 1 Computing Science and Mathematics, University of Stirling, Stirling, Scotland, FK9 4LA, UK; 2 School of Informatics, University of Edinburgh, Edinburgh, Scotland, EH8 9AB, UK

## 

Neuronal computations come with a certain metabolic cost. Consequently, the brain’s limited energy supply imposes constraints on its information processing capability. Neural modulators, such as nitric oxide (NO), alter neural network properties in response to activity, and consequently will likely affect energy usage as well as function. Based on experimental data of the effects of NO in the auditory brainstem, we use a computational model to address the question as to what extent neuronal modulation by NO could change the efficiency or the metabolic cost of neuronal transmission.

We have built a three component model (pre-synaptic, post-synaptic and action potential initiation zone) of the calyx of Held and its target, the medial nucleus of the trapezoid body (MNTB) principle neuron, to estimate the metabolic efficiency in this model system. Model parameters are fit such that data from whole cell recordings is reproduced, and models are compared between a baseline condition with low levels of NO, and following conditioning with evoked activity that increases NO levels. Raised NO modulates both synaptic short term depression and cell excitability through re-balancing of K+ conductances.

The synapse model exhibits short term depression, which was implemented using a standard depletion model. Differences between the unconditioned (control) and conditioned (NO) model were implemented as changes in release probability and vesicle recycling rate. The MNTB neuron is represented by a single compartment, Hodgkin-Huxley type model. The ionic conductances were identified from ventral cochlear nucleus bushy cells [1] and MNTB principal neurons [2]. The channel densities were set to reproduce the control and NO-modulated conditions in the MNTB neurons [3]. For each compartment, metabolic cost was scaled to match the data from synaptic activity or action potential firing during very low frequency stimulation [4] and calculated in the same way for the higher frequency activity commonly seen at this synapse. The efficiency of action potentials was calculated based on the overlap of the underpinning sodium and potassium currents and the total area of the sodium current [5].

Our results show that in the case of NO modulation, the MNTB neuron model has a higher efficiency for AP generation (58.5%) than in control conditions (49.5%). For high frequency stimulation, NO modulation very slightly increases the energy cost per vesicle released, (49.5*10^3^ ATP molecules), compared to the control case (48.6*10^3^ ATP molecules). Depression is less under NO, so that more vesicles are released at the end of a high frequency train than in control conditions. We have quantified the higher energetic cost implied by the higher frequency at which the principle neuron is able to fire for the NO case.
